# Two Cases of Fatal Injury of the Head

**Published:** 1852-08

**Authors:** Robert Durrett

**Affiliations:** Resident Graduate of the Louisville Marine Hospital; Louisville


Art. II.—*Two Cases of Fatal Injury of the Head.* By Robert Durrett, M. D., Resident Graduate of the Louisville Marine Hospital, for 1851.

Miller in his Practice of Surgery says, that "between pure examples of concussion and compression of the brain, there is no difficulty in drawing a sufficiently broad distinction in practice as well as in theory. The one, a case of syncope; the other, one of coma. In concussion, the symptoms immediate; insensibility incomplete; the organs of special sense capable of being roused; the muscles contractile, and the limbs under strong stimulus, undergoing movement; the breathing soft and gentle; the pupils not uniformly dilated, though insensible to light; the pulse rapid, small, indistinct, perhaps for a time imperceptible; vomiting; no involuntary evacuations; the skin cold, pale, and shrunk. In compression, the symptoms not necessarily immediate; insensibility complete; the organs of special sense incapable of being roused; the muscles relaxed, paralyzed; the limbs motionless, until cessation of the state of compression, and advance of the inflammatory process; breathing labored, slow, and snoring; the pupils dilated and insensible; the pulse slow, distinct, perhaps full, sometimes intermittent; no (or seldom) vomiting; feces passed involuntarily; retention or dribbling urine; the skin warm, and often bedewed by perspiration."

The history of the two following cases does not accord with the above quotation.

John Convoy, aged thirty, a marine, was admitted into the surgical ward of Louisville Marine Hospital, March 23d, 1852. He gave the following very unsatisfactory account of himself, to wit: That he had been drinking for several days, and either fell and injured himself or received blows from an unknown source. Upon examination, found exquisite tenderness over the spine between the shoulders, which was relieved by the application of wet cups. The patient complained a little of his head, tongue was good, pulse about 60, natural in volume, pupils susceptible to light. I supposed the patient to be laboring under the effects of a debauch. Ordered a purge.

March 24th. Patient still unable to tell how he was hurt, but thinks that he was struck by some one whilst drunk; seems to answer all questions propounded intelligibly, complains of constant and distressing head ache; no marks of external injury. Ordered patient's hair trimmed, and cold water applied to the head.

March 25th. Patient sleeps almost constantly, sings and talks in sleep; when aroused, which is done with difficulty, answers questions directly; pulse 70, pupils susceptible to light. Having heard to-day through friends of the patient that he had been struck by the mate of the boat several times, supposed the patient laboring under inflammation of the brain, the result of concussion caused by the infliction of the blows. Ordered submur. hydrar., grs. ij, every three hours, the continuation of cold water to the head.

March 26th. No perceptible change, except slight opisthotonos when patient attempts to move.

March 27th. Opisthotonos continues; patient walks across the ward to the privy.

March 28th. Opisthotonos continues to increase in severity, pulse less full, more frequent, skin dry, rather warm. Ordered, a blister to the back of head and neck.

March 29th. Stopped mercury; patient ptyalised; slight delirium, decided opisthotonos, pulse 100, intermitting; bowels moved by medicine.

March 30th. Patient died with the severest convulsion I have ever seen. On a post-mortem examination, found a fracture almost throughout the entire extent of the lambdoidal suture on the right side; between two and three ounces of coagulated blood immediately beneath the fracture, between the dura mater and cranium ; over an ounce of serum in the right ventricle ; lymph at the base of the brain, and softening of the spinal cord. The patient's intellect remained unimpaired until within a few hours of his death, showing that the cerebellum is capable of sustaining the gravest injury without materially affecting the mind. It is very difficult, and in some cases almost impossible, to make out a correct diagnosis in injuries or diseases of the brain. Many of the most prominent symptoms of compression may be present during life, and yet autopsies reveal none of the pathological changes, which the symptoms are supposed to indicate, as in the following case.

Allen Smith was admitted into the Hospital, March 15, 1852, one hour after the reception of two incised, and one contused wound upon the head, which felled him insensible to the ground. The patient had retention of urine, dilated pupils, warm extremities, partial unconsciousness, involuntary fecal discharges and stertorous respiration, for several hours before death. A careful examination of his head did not enable us to account for his death, which occurred on the 17th of March, thirty-six hours after injury. We found considerable engorgement of the venous system, and some serum in the ventricles ; but not more than we usually find in autopsies—not so much as we invariably find in persons who die of *mania a potu.* I give these particulars because Smith was a man of very intemperate habits, and at the time he received the blows was, without doubt, the subject of delirium tremens.

Louisville, 1852.

